# Medial Congruent and Medial Pivot Inserts in Total Knee Arthroplasty: A Scoping Review

**DOI:** 10.3390/medicina61050844

**Published:** 2025-05-03

**Authors:** Francesco Romano, Roberto Rossi, Umberto Cottino, Matteo Bruzzone, Francesco Pirato, Federica Rosso

**Affiliations:** 1 Department of surgery, Università degli Studi di Torino, Via Po 8, 10100 Torino, Italy; francescoromano879@gmail.com (F.R.); pirato.francesco@gmail.com (F.P.); 2 AO Ordine Mauriziano, Department of Orthopaedics and Traumatology, University of Torino, Largo Turati 62, 10128 Torino, Italy; rossir@fastwebnet.it (R.R.); umberto.cottino@gmail.com (U.C.); matteobruzzone@yahoo.it (M.B.)

**Keywords:** total knee arthroplasty, medial pivot, medial congruence, outcomes

## Abstract

*Background and Objectives*: Total knee arthroplasty (TKA) is one of the most common medical procedures worldwide. However, 10 to 20% of patients are still dissatisfied despite implants and surgical technique advancements. Recently, several medial-stabilized TKAs have been developed in attempts to replicate the native kinematics of the knee. The aim of this scoping review on medial-stabilized TKA inserts—medial congruent (MC) and medial pivot (MP)—is to focus on their clinical outcomes and the role of the posterior cruciate ligament (PCL), aiming to systematically map the existing research and highlight current knowledge gaps. *Materials and Methods*: A search of the PubMed, Embase and Cochrane databases was performed to identify relevant studies on the kinematics and outcomes of medial pivot (MP) or medial congruent (MC) inserts. The following Mesh terms were used in combination with the Boolean operators “AND” and “OR”: “total knee arthroplasty”, “total knee replacement”, “medial pivot”, “medial congruence”, “outcomes” and “kinematic”. Original studies reporting on clinical outcomes assessed with validated patient-reported scales, surgical techniques and reoperation rates for any reason with a minimum follow-up of 18 months were included. *Results*: A total of 39 articles met the inclusion criteria, accounting for 6143 total knee replacements. The overall reoperation-free survivorship rate was 98.4% (6047 out of 6143 knees) at a weighted average follow-up of 6.3 years (range 1.5–15.2 years, SD 0.7). Both MP and MC inserts demonstrated good outcomes, with no differences between groups. Few studies evaluated the role of the PCL in MP and MC inserts, with no differences in terms of clinical outcomes between retaining and sacrificing the PCL. *Conclusions*: MS-TKA demonstrated good outcomes in the literature independently of the specific design (medial pivot or medial congruent). Different possible biases may be present when evaluating the outcomes of these inserts, including different types of alignment and soft tissue balancing philosophies.

## 1. Introduction

**Figure 1:**
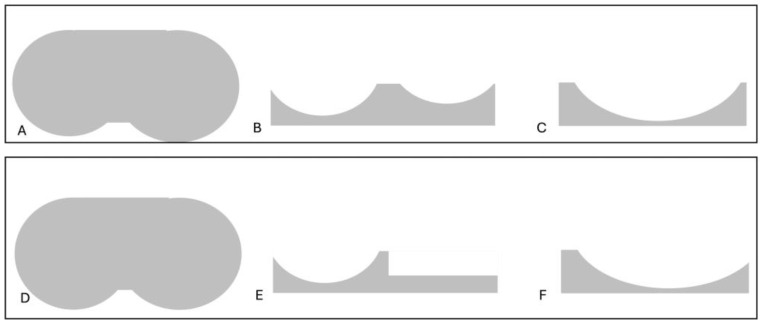
In the upper part of the figure, an MC insert is shown: (**A**) Axial view demonstrating the anatomic asymmetric shape of the tibial plateau with a larger medial surface. (**B**) AP view demonstrating a more concave medial surface for a more constrained medial compartment compared to the lateral one. (**C**) Lateral view showing the deep anterior and posterior lips to achieve AP stability. In the lower part of the figure, an MP insert is shown. (**D**) Axial view demonstrating the symmetric shape of the tibial plateau. (**E**) AP view demonstrating a more concave medial surface and a flat lateral one, reproducing the “medial pivot” kinematics of the knee. (**F**) Lateral view showing the deep anterior lip to achieve AP stability.

Total knee arthroplasty (TKA) is a widely performed procedure for treating end-stage knee osteoarthritis [1]. However, up to 20% of patients are dissatisfied due to persistent pain, instability or unnatural knee motion [2]. Different factors may be related to this dissatisfaction rate, including the surgical technique, the type of implant and the post-operative protocol. To improve the rate of satisfied patients, surgeons may “personalize” TKA either through the surgical technique, by choosing new alignment philosophies, or the implant, by choosing new bearings. Different studies have confirmed that normal knee kinematics follow a “medial pivot” concept, with the medial compartment being relatively “fixed” and the lateral compartment performing a physiological combination of rolling back and translational movements during flexion [3,4]. For this reason, medially conforming inserts have recently been introduced with the goal of recreating “medial pivot” kinematics [5]. Particularly, two bearings with slightly different kinematics can be distinguished. In the so-called “medial pivot” design (MP), the insert has a peculiar shape which includes a concave medial compartment providing a large contact surface, and a flatter lateral surface facilitating the femoral rollback. MP bearing is usually associated with a single-radius femoral component (SR) that closely reproduces a true ball-and-socket mechanism [6,7]. Conversely, the “medial congruent” insert (MC) is highly congruent on the medial side with a more posterior dwell point, resulting in a good reproduction of the knee kinematics. This type of insert is associated with a J-curve or multi-radius femoral condyle (MR). Both MP and MC inserts have demonstrated similar biomechanical properties, even though they have slightly different designs. Particularly, in MP inserts, tibial rotation close to the native knee has been demonstrated, in association with good AP stability and more natural joint kinematics [8,9]. Furthermore, medial-stabilized implants, in comparison to the UC design, demonstrated similar total tibio-femoral contact forces over the whole knee flexion movement but with a more favorable relative contact pressure distribution and lower peak contact pressures in the MP design, which may suggest a lower rate of wear and potentially enhances implant’s longevity [9]. Similarly, the MC design demonstrated good tibial external rotation in maximum extension, confirming the restoration of the screw-home mechanism with good anteroposterior stability and the avoidance of mid-flexion instability [10]. Figure 1 summarizes the different insert characteristics.

Good survivorship has been demonstrated in both MP and MC implants [11,12], with similar or better clinical outcomes and patient satisfaction compared to posterior stabilized TKA at short-term follow-up [13,14,15,16,17]. Furthermore, both an MP and an MC insert can be implanted while sacrificing or respecting the posterior cruciate ligament (PCL), with conflicting results reported in the literature [18,19,20,21,22,23].

The aim of this scoping review is to summarize the available literature on these two closely related MS-TKA inserts, highlighting their differences in terms of outcomes. Furthermore, an analysis of the differences between respecting or sacrificing the PCL with these inserts will also be performed.

## 2. Materials and Methods

### 2.1. Literature Search

Extensive research in PubMed, Embase and Cochrane databases was performed to identify relevant studies on kinematics and outcomes of medial pivot (MP) or medial congruent (MC) inserts. The articles in this study included all English-written papers published up to December 2024. The following Mesh terms have been used in combination with Boolean operators “AND” and “OR”: “total knee arthroplasty”, “total knee replacement”, “medial pivot”, “medial congruence”, “outcomes” and “kinematic”. The search yielded 369 results. The papers that met inclusion criteria were original studies, describing outcomes of medial-stabilized (MS) implants (either MP or MC) in primary total knee arthroplasty (TKA), in which there was a description of the surgical technique and re-operation rate for any reason, with a minimum follow-up of 18 months. Case reports, review articles, expert opinions, letters to editors, biomechanical studies, investigations involving animals, cadaver studies, in vitro research, book chapters as well as studies published in a language other than English were excluded. Titles and abstracts were screened by two authors (F.R. and F.R. blinded for review) to identify the included studies [6,7,14,15,16,17,18,19,20,21,22,23,24,25,26,27,28,29,30,31,32,33,34,35,36,37,38,39,40,41,42,43,44,45,46,47,48,49,50]. Disagreements on study selection were resolved by discussion with a third reviewer (F.P.) when necessary.

Different data were collected for every article, including the year of publication, level of evidence of the study, number of patients, length of follow-up, number of patients dead or lost to follow-up, diagnosis, age, Body Mass Index (BMI), sex, implant system and features, surgical technique (posterior cruciate ligament resected or preserved, patella resurfaced or not, alignment), re-operation for any reason and patient-reported outcomes. Data from each eligible article was independently collected by two reviewers (F.R. and F.R., blinded for review). Any disagreements were addressed by consultation with a third reviewer (F.P.). All study data were collected using Microsoft Excel (Microsoft Corp., Redmond, WA, USA). Table 1 summarizes the evaluated studies.

After an initial overall analysis of MS-TKA outcomes, a detailed evaluation of MP or MC outcomes was performed, as well as a subgroup analysis evaluating the differences between PCL-retained and PCL-scarified implants.

### 2.2. Statistical Analysis

Categorical variables are presented as percentages. Continuous variables are presented as means, weighted by sample size, with the range between minimum and maximum values and the standard deviation.

## 3. Results

### 3.1. Study Selection

**Figure 2:**
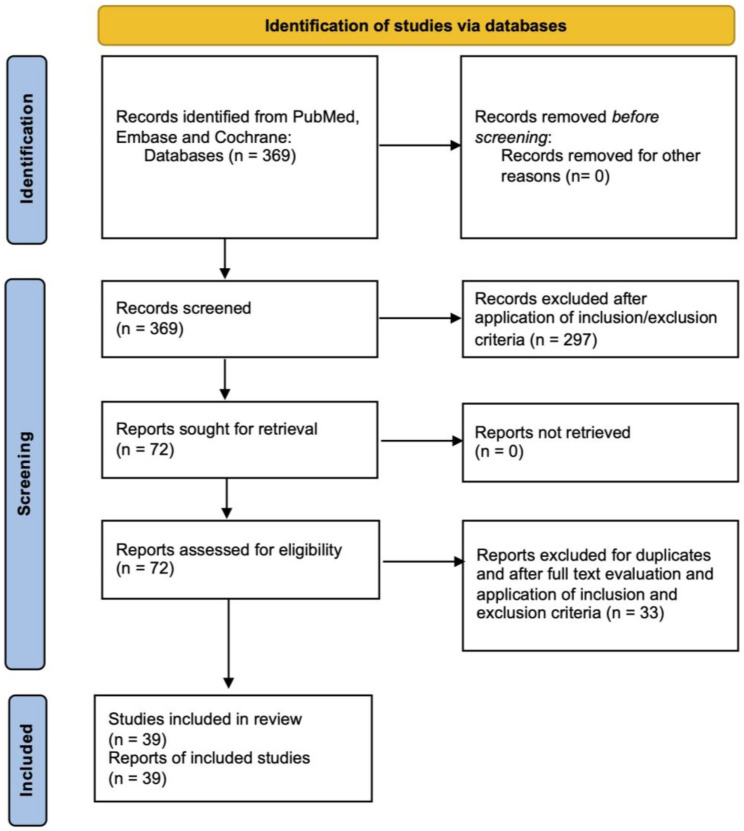
PRISMA diagram.

The selection process is illustrated in Figure 2. The literature search yielded a total of 369 references. After removing duplicate papers and applying inclusion and exclusion criteria, 72 articles were assessed for eligibility. After full-text evaluation, 39 studies were included in this scoping review [6,7,14,15,16,17,18,19,20,21,22,23,24,25,26,27,28,29,30,31,32,33,34,35,36,37,38,39,40,41,42,43,44,45,46,47,48,49,50].

### 3.2. Demographic Data

A total of 697 total knee replacements in 5887 patients were performed in the 39 evaluated studies. Accounting for patients lost to follow-up, the final analysis included 6143 medial stabilized total knee arthroplasties (MS-TKAs). The weighted average age was 69.9 years (range 55–78 years, SD 3.3) and the weighted average BMI was 28 kg/m^2^ (range 23.4–34.6, SD 1.2), with 66.1% of female patients. Indication for TKA was knee osteoarthritis in 94.6% of patients, rheumatoid arthritis in 4.6% and 0.8% were indicated for other reasons, such as osteonecrosis. The average follow-up was 6.3 years (range 1.5–15.2 years, SD 0.7). There were 80.2% of medial pivot implants (n = 4965) and 19.2% of medial congruent implants (n = 1178). The different types of implants utilized are summarized in Table 2.

In twenty-three studies, TKA was implanted with a mechanical alignment, either with gap balancing or measured resection techniques [6,7,15,16,17,19,20,21,22,24,25,27,28,31,32,34,37,39,41,43,44,48,50]. In three studies, kinematic alignment philosophy was used [14,23,49]. One randomized controlled trial specifically compared the clinical outcomes of mechanically versus kinematically aligned MP- TKA [47]. One prospective observational study described a functional alignment with a “ligament-driven” technique [18]. Different alignments are summarized in Table 3.

### 3.3. General Outcomes

Thirty-three studies [6,7,14,15,16,17,19,20,21,22,23,24,25,26,27,28,30,32,33,34,35,36,38,39,40,41,42,43,44,45,46,48,50] reported on the range of motion of 5447 TKAs, with a weighted average of 115.2° (range 104°–132.1°, SD 5.8). In thirty-two studies (5202 TKAs) [6,7,14,17,19,21,22,23,24,25,26,27,29,30,32,33,34,35,36,37,38,40,41,42,43,44,45,46,47,48,49,50] the Knee Society Knee Score was used to evaluate the clinical outcomes, in twenty-six studies (4305 TKAs) [6,7,17,19,21,23,24,26,30,32,33,34,35,36,37,38,41,42,43,44,45,46,47,48,49,50] the functional Knee Society Score was used, in eleven studies (1601 TKAs) [6,7,15,17,18,20,24,38,45,47,50] the Oxford Knee Score and in fourteen studies (3102 TKAs) [21,24,28,31,32,36,37,38,42,43,45,47,48,49] the Western Ontario and McMaster University (WOMAC) score were used. The post-operative Forgotten Joint Score was used only in sixteen studies for 3322 TKAs [7,14,16,18,20,21,22,23,24,27,36,39,41,46,47,48]. A significant improvement from pre-operative to post-operative scores was demonstrated in all studies. Average scores are reported in Table 4 and Table 5.

### 3.4. Reoperation

The overall reoperation-free survivorship rate was 98.4% (6047 out of 6143 knees) at a weighted average follow-up of 6.3 years (range 1.5–15.2 years, SD 0.7), excluding patients who were deceased for reasons unrelated to surgery or lost to follow-up. The overall reintervention rate was 1.6%, mainly due to periprosthetic fractures (0.4%), aseptic loosening (0.2%) and infections (0.7%). The average reintervention rate was 1.3% (range 0–5.8%, SD 1.5).

### 3.5. Medial Pivot

A medial pivot implant was evaluated in thirty studies [6,7,14,15,16,17,19,21,29,30,31,32,33,34,35,36,37,38,39,40,41,42,43,44,45,46,47,48,49,50], for a total of 4965 MP-TKAs, representing 80.8% of the total sample. The average follow-up was 6.8 years (range 2–15.2, SD 0.7). Table 6 summarizes these studies.

**Figure 3:**
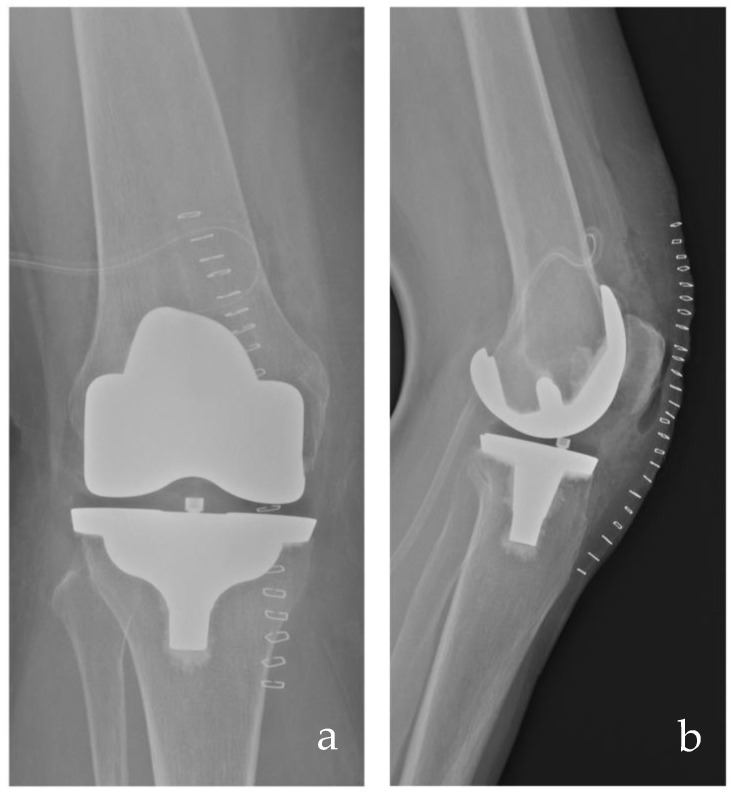
Post-operative anteroposterior (**a**) and lateral (**b**) radiographs of a medial pivot TKA from the senior author’s case series.

The medial pivot TKA was designed to replicate the kinematics of the natural knee joint, trying to enhance patient comfort and satisfaction while providing a more “natural” knee kinematics [5,8,9]. A key aspect of the MP implant is the asymmetry between its medial and lateral compartments. The medial compartment features high congruence and a large contact surface, contributing to stability and pivoting kinematics. In contrast, the lateral compartment has a flat surface allowing a sliding motion of the lateral femoral condyle while enabling tibial internal rotation and femoral external rotation during knee flexion. Anteroposterior stability is provided by the anterior and posterior lips, which act as replacements for the anterior and posterior cruciate ligaments, respectively, justifying the term “medially stabilized implants.” This type of insert is usually associated with a single-radius femoral component, creating the so-called “ball-in-socket” mechanism. This association between MP insert and single radius femoral component effectively prevents anterior femoral sliding, known as paradoxical movement, which is often observed in conventional multi-radius implants based on the four-bar link theory [8,9]. Figure 3 shows a medial pivot implant.

General outcomes of medial pivot TKA have been evaluated in nineteen retrospective studies [6,7,19,21,31,32,33,35,36,38,39,40,41,42,44,46,48,49,50], with a total of 3736 TKAs. These studies described good outcomes at a weighted average follow-up of 6.3 years (range 2–13.4, SD 0.6) (Table 5). Xiang et al. evaluated more than 1100 patients, the largest case series published, with good to excellent clinical outcomes and a mid-term cumulative survival rate of 99.2% (95% CI, 99.6–98.4%). Karachalios et al. have reported the longest follow-up among retrospective observational studies, with a success rate of 97.3% (95% CI 96.7 to 97.9) at 15 years with revision for any reason as the end point. One study specifically investigates the results of the medial pivot TKA in patients affected by rheumatoid arthritis [50], with satisfactory clinical outcomes at a minimum follow-up of three years. In two studies, a medial pivot implant was compared with a rotating-platform mobile-bearing implant [32,48] with no differences in clinical outcomes, even though the findings of Shakya et al. described better FJS and functional KSS for medial pivot implants at a 7.1-year average follow-up.

Outcomes of medial pivot implants compared to other types of inserts were evaluated in six randomized controlled trials. Particularly, four studies compared an MP-TKA with a posterior stabilized TKA (PS-TKA) [14,15,16,17], one study compared the results of medial pivot implants implanted with mechanical or a kinematic alignment [47] and one study evaluated the outcomes of medial pivot compared to a cruciate-retaining (CR) rotating platform [43]. Batra et al. reported increased KSS-Satisfaction and Expectation scores at the four-year follow-up for MP-TKAs compared to PS-TKAs. In the study by Chang et al., no significant differences in outcomes were observed at the two-year follow-up between MP or PS implants in association with a single radius femoral component. Furthermore, Kulshrestha et al. demonstrated significantly better performance at the Direct Observation of Procedural Skills (DOPSs) in patients with an MP insert compared to those with PS implants. Ettinger et al. reported that MP-TKA associated with kinematic alignment had a superior joint awareness score (Forgotten Joint Score) as well as higher expectation and satisfaction at 1-year and 2-year follow-up compared to those implanted with a mechanical philosophy, especially in cases of varus morphotype. Conversely, Kim et al. reported superior clinical results with a CR mobile bearing implant compared to an MP-TKA with a minimum 11-year follow-up.

Five prospective observational studies described the clinical outcomes of medial pivot TKA [29,30,34,37,45]. Macheras et al. reported on the longest follow available in the literature (15.2-years average follow-up, range 15–17 years) with a cumulative survivorship of 98.8% (95% confidence interval: 97.6–100%) at 17 years. During the follow-up period, four cases (1%) required revision: three for persisting anterior knee pain (requiring patella resurfacing) and one case for a periprosthetic tibial fracture.

Two studies focused on the comparison between medial pivot TKA with a medial congruent TKA [6,7], with no statistically significant differences in short-term clinical outcomes at the two-year follow-up. However, the authors demonstrated that J-curved implants had a greater post-operative range of motion (ROM). Additionally, Vecchini et al. described a higher Forgotten Joint Score in patients with medial pivot (MP) implants compared to medial congruent (MC) ones.

### 3.6. Medial Congruent

A medial congruent bearing was evaluated in eleven studies [6,7,18,20,22,23,24,25,26,27,28] with 1178 MC-TKAs (19.2% of the total sample) at a weighted average follow-up of 3.8 years (range 1.5–6, SD 0.3).

**Figure 4:**
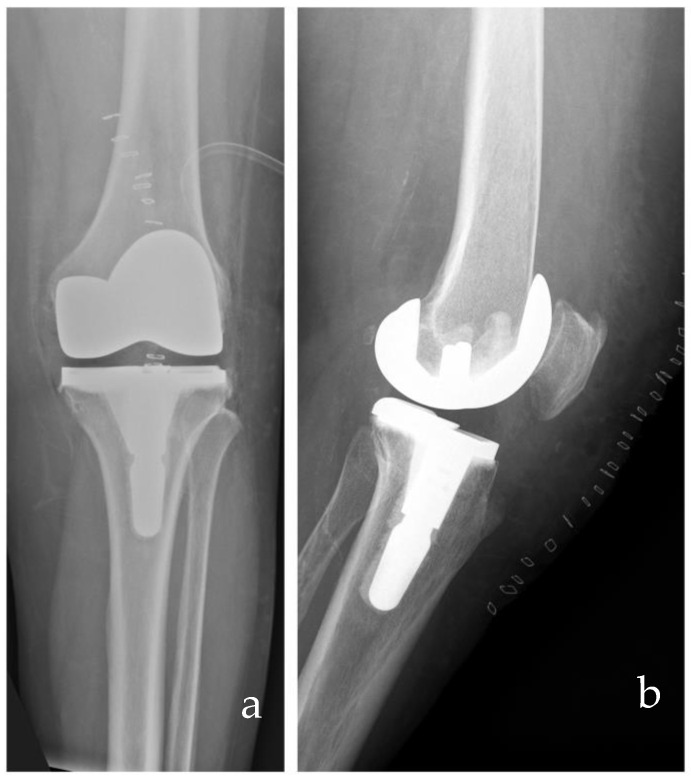
Post-operative anteroposterior (**a**) and lateral (**b**) radiographs of a medial congruent TKA from the senior author’s case series.

Medial congruent inserts have been recently introduced with a design mainly based on the kinematics of ultracongruent (UC) implants. They are usually characterized by a highly congruent medial side, with a more posterior dwell point, allowing the femoral roll back, and an anterior and posterior lip for anteroposterior stability. These inserts are usually associated with the cruciate-retaining J-curved femoral component, and they were initially designed for both retaining or sacrificing the posterior cruciate ligament. Figure 4 shows an X-ray of an MC-TKA.

Faschingbauer et al., in their cadaveric study on one of these implants (Persona^®^ Zimmer Biomet, Warsaw, IN, USA), compared the kinematics of ultracongruent (UC), cruciate-retaining (CR) and medial congruent (MC) inserts. In this study, the MC insert demonstrated the least degree of femoral rollback, tibio-femoral rotation and single bony rotations, exhibiting a pronounced paradoxical roll forward, but still showing a greater degree of constraint than the UC and CR inserts [53].

Two randomized clinical trials have been conducted with MC-TKA [20,26]. Nishitani et al. have evaluated the same J-curved implant with two different inserts, one asymmetrical (MC) and one symmetrical, and reported no significant differences in clinical outcomes or post-operative ROM at the two-year follow-up.

Furthermore, seven retrospective studies have investigated the overall outcomes of MC-TKAs [6,7,23,24,25,27,28]. Among these studies, two studies previously mentioned [6,7] compared the outcomes of MP and MC bearings while two studies have analyzed the clinical outcomes of MC-TKAs in valgus knee osteoarthritis (OA) [27,28], with favorable results at short-term follow-up. Only one retrospective observational study evaluated the clinical outcomes of an MC-TKA implanted with a kinematic alignment philosophy, with good results [23]. Table 7 summarizes the outcomes of these studies.

### 3.7. The Role of Posterior Cruciate Ligament

Even if the MS-TKA has been designed to overcome the stabilizing role of the PCL, surgeons can decide to either preserve or sacrifice the posterior cruciate ligament. However, the literature lacks standardized guidelines supporting an evidence-based decision. In the evaluated papers, three retrospective studies compare MP-TKA with or without the PCL [19,21,23], with no differences in clinical outcomes between the two groups. In the RCT by Budhiparama et al. [20], the MC-TKA with or without PCL preservation was compared in simultaneous bilateral TKA, with no significant differences in range of motion or clinical outcomes at two-year follow-up. The clinical outcomes and survival rates of a medial congruent TKA with or without PCL were compared in two prospective observational studies [18,22]. Rossi et al. analyzed the outcomes of 165 TKRs divided into two groups: PCL-preserved and PCL-sacrificed implants. Each group underwent the same surgical technique, performed by a single surgeon, with a slight reduction in the tibial slope in the PCL-sacrificing group in order to balance the flexion gap. The results showed no significant differences in clinical outcomes at mid-term follow-up. Instead, Rajgopal et al. compared 60 simultaneous bilateral MC-TKA with or without the PCL, reproducing in all knees the native tibial slope. Their findings indicated that the PCL-sacrificed group had a greater post-operative ROM, while the PCL-preserved group had a higher Knee Society Score (KSS) at an 18-month follow-up.

In medial stabilized implants, preservation of the PCL may result in more physiological knee kinematics [18,19,20,21,22,23]. However, the excision of the PCL, with a consequent increase in flexion gap may be correlated with a greater range of motion, as evidenced by Rajgopal et al. While the effects of PCL on the flexion gap are more straightforward and can be assessed intraoperatively with tests such as the anterior lift-off, determining the influence of PCL on knee biomechanics is difficult. Nedopil et al. suggested that the PCL plays a critical role in driving tibial internal rotation: its tension facilitates internal rotation of the tibia during knee flexion, which subsequently reduces the Q-angle optimizing the extensor mechanism [54]. On the other hand, Moewis et al. [55] showed that the retention of PCL alone is insufficient to reduce the anterior translation of the femur in the early knee flexion and to reproduce the lateral femoral roll-back. Howell et al. [56] have underlined that a PCL-retaining technique combined with kinematic alignment should be utilized even in cases of valgus deformity, where the PCL is tight compared to neutral or varus-aligned knees. However, Indelli et al. [27] reported good clinical outcomes in valgus knee OA treated with an MC-TKA performing a posterior capsular release in 72% of the cases, without recording any case of post-operative instability.

## 4. Discussion

Medial stabilized implants are designed to mimic native knee kinematics while preventing the paradoxical anterior sliding of the femoral condyles in TKA, following the findings by Freeman and Pinskerova [3,4] that first described the pivot mechanism in non-arthritic knees. Various manufacturers have developed their own implants based on this philosophy to ideally enhance patient satisfaction, eliminate residual pain and fulfill patients’ pre-operative expectations. Consequently, research about medial stabilized total knee arthroplasty is expanding, with different papers being published in recent years. However, there is some confusion between the different types of insert (medial congruent or medial pivot) and the different types of femoral components (ball-in-socket or J-curve). The primary aim of this scoping review was to comprehensively analyze different studies, focusing on the differences between medial pivot and medial congruent inserts, including clinical outcomes and also considering other variables related to surgical technique, such as alignment and soft tissue balancing philosophies.

A total of 39 primary studies were included in this scoping review. There are numerous studies supporting good clinical results of medial pivot TKA. On the other hand, the literature on medial congruent TKA is less extensive, but it similarly shows good results. There are few studies confirming the long-term survivorship of medial pivot implants [33,37,38,43,45,46], while only mid-term studies have been conducted on medial congruent TKA’s [18,24] and further research is needed to assess long-term outcomes and survival rates. Medial congruent implants have been developed more recently, which may contribute to the limited number of studies available. Few studies directly compare MP and MC inserts, reporting no significant differences in clinical outcomes [6,7]. However, in most studies, not only different inserts but also different alignment philosophies are described (Table 1 and Table 3), with possible biases. Surgeons should be aware that MP and MC inserts have different biomechanical characteristics, which may yield similar kinematics and consequently, similar outcomes, even if different alignment philosophies are adopted. A limited number of studies analyzed the outcomes of kinematic alignment in association with medial stabilized TKA [14,23,47,49], highlighting another lack of evidence in the literature. The randomized trial conducted by Ettinger et al. concluded that patients undergoing MP TKA with restricted kinematic alignment may report improved joint awareness (FJS) and higher satisfaction levels (KSS satisfaction and expectation scores) compared to MP TKA associated with mechanical alignment at short-term follow-up. The authors suggested that integrating the two aforementioned techniques, developed in order to reproduce the native knee kinematics—rKA, which emphasizes the preservation of soft tissues, and the MP implant that ideally maintains pivot kinematics—may lead to better clinical outcomes.

The choice between MP and MC bearings cannot be evidence-based due to a lack of strong clinical evidence, but it is still up to the surgeon’s preference, similar to the opportunity to sacrifice or retain the PCL with the same bearing surfaces. The role of the posterior cruciate ligament in MP or MC inserts remains debated. Current studies agree that retaining the PCL is unnecessary with a medial-stabilized design, given the anterior–posterior stability achieved by the insert itself, suggesting that both preserving or sacrificing the PCL can be effective and, again, a surgeon’s preference [18,19,20,21,22,23]. However, if the PCL is sacrificed, tibial slope correction should be performed to balance the flexion gap. Only Rajgopal et al. reported significant differences between the PCL-retained and PCL-sacrificed groups, demonstrating higher KSS-2011 and FJS-12 and a gait pattern closer to normal at short-term follow-up in the retained group. Similarly, Budhiparama et al. concluded that retaining the PCL favors the tibia’s internal rotation during flexion, which optimizes patellofemoral tracking, though this results in a tighter flexion gap. Hu et al. concluded that preserving the PCL can be beneficial in cases where the extension and flexion gaps are balanced, as this can enhance patient proprioception; additionally, the authors suggested preferring a slight varus alignment to sustain medial tension and ensure knee stability.

This scoping review has some limitations. First, it only focuses on studies evaluating clinical outcomes, excluding biomechanical studies, which may offer crucial insights into the kinematics of total knee arthroplasty (TKA). Furthermore, it was not possible to directly compare the outcomes between MP and MC designs due to the different numerosity of the studies. However, MC designs have been introduced more recently than MP implants, and further studies are needed in order to reach long-term outcomes and significant clinical evidence.

## 5. Conclusions

Both MP and MC are effective in reproducing knee kinematics, with good outcomes. However, there is no available evidence to guide the decision-making process in sacrificing or retaining the PCL in these implants. High-quality studies (i.e., randomized trials) with long follow-ups may be necessary to better understand the role of the PCL and tibial slope in this type of insert and to evaluate their long-term survivorship.

## Figures and Tables

**Table 1:** Summary of the studies.

Author	Year	Study Type	Sample (n)	Follow Up (y)	Insert Type	Alignment	PCL
Anderson et al. [29]	2010	Observational Prospective	238	5.4	Medial Pivot	Unclassified	unclassified
Bae et al. [19]	2011	Observational Retrospective	137	3.9	Medial Pivot	Mechanical	preserved: 67 sacrificed: 70
Batra et al. [15]	2021	Randomized Controlled Trial	53	4	Medial Pivot	Mechanical	sacrificed
Brinkman et al. [37]	2014	Observational Prospective	50	10	Medial Pivot	Mechanical	preserved: 27 sacrificed: 23
Budhiparama et al. [20]	2023	Randomized Controlled Trial	66	2.7	Medial Congruent	Mechanical	preserved: 33 sacrificed: 33
Cacciola et al. [24]	2020	Observational Retrospective	351	5.5	Medial Congruent	Mechanical	unclassified
Chang et al. [17]	2021	Randomized Controlled Trial	45	2	Medial Pivot	Mechanical	sacrificed
Chinzei et al. [44]	2014	Observational Retrospective	85	7.8	Medial Pivot	Mechanical	sacrificed
Choi et al. [32]	2017	Observational Retrospective	49	5.3	Medial Pivot vs. Rotating Platform Mobile Bearing	Mechanical	sacrificed
Dehl et al. [33]	2018	Observational Retrospective	50	9.5	Medial Pivot	Unclassified	unclassified
Ettinger et al. [47]	2024	Randomized Controlled Trial	98	2	Medial Pivot	Mechanical vs. Kinematic	unclassified
Fan et al. [34]	2010	Observational Prospective	58	5.4	Medial Pivot	Mechanical	sacrificed
Giustra et al. [23]	2022	Observational Retrospective	64	2.4	Medial Congruent	Kinematic	preserved: 35 sacrificed: 29
Hu et al. [21]	2023	Observational Retrospective	252	8.7	Medial Pivot	Mechanical	preserved: 84 sacrificed: 168
Indelli et al. [27]	2023	Observational Retrospective	79	3.6	Medial Congruent	Mechanical	unclassified
Indelli et al. [6]	2020	Observational Retrospective	100	2	Medial Congruent vs. Medial Pivot	Mechanical	sacrificed
Iwakiri et al. [28]	2022	Observational Retrospective	162	3.8	Medial Congruent	Mechanical	unclassified
Karachalios et al. [38]	2016	Observational Retrospective	251	13.4	Medial Pivot	Unclassified	preserved: 183 sacrificed: 68
Katchky et al. [31]	2019	Observational Retrospective	81	5.3	Medial Pivot	Mechanical	unclassified
Kim et al. [43]	2017	Randomized Controlled Trial	182	12.1	Medial Pivot	Mechanical	sacrificed
Kulshrestha et al. [16]	2020	Randomized Controlled Trial	36	2	Medial Pivot	Mechanical	unclassified
Macheras et al. [45]	2017	Observational Prospective	347	15.2	Medial Pivot	Unclassified	preserved: 184 sacrificed: 163
Malhotra et al. [50]	2021	Observational Retrospective	36	3.72	Medial Pivot	Mechanical	sacrificed
Mannan et al. [30]	2009	Observational Prospective	172	6	Medial Pivot	Unclassified	unclassified
Nakamura et al. [25]	2018	Observational Retrospective	45	2	Medial Congruent vs. Cruciate Retaining	Mechanical	preserved
Nishitani et al. [26]	2018	Randomized Controlled Trial	33	2	Medial Congruent vs. Cruciate Retaining	Unclassified	unclassified
Rajgopal et al. [22]	2023	Observational Prospective	120	1.5	Medial Congruent	Mechanical	preserved: 60 sacrificed: 60
Rossi et al. [18]	2024	Observational Prospective	165	6	Medial Congruent	Functional	preserved: 80 sacrificed: 85
Schmidt et al. [40]	2014	Observational Retrospective	365	5.3	Medial Pivot	Unclassified	sacrificed
Scott et al. [14]	2022	Randomized Controlled Trial	88	2	Medial Pivot vs. Posterior Stabilized	Kinematic	sacrificed
Shakya et al. [48]	2022	Observational Retrospective	52	7.3	Medial Pivot	Mechanical	sacrificed
Shi et al. [39]	2020	Observational Retrospective	290	6.7	Medial Pivot	Mechanical	unclassified
Sosio et al. [49]	2023	Observational Retrospective	55	2	Medial Pivot	Kinematic	sacrificed
Ueyama et al. [46]	2020	Observational Retrospective	257	10.1	Medial Pivot	Unclassified	sacrificed
Ueyama et al. [41]	2022	Observational Retrospective	153	5	Medial Pivot	Mechanical	sacrificed
Vecchini et al. [7]	2023	Observational Retrospective	89	2.1	Medial Congruent vs. Medial Pivot	Mechanical	sacrificed
Vecchini et al. [35]	2012	Observational Retrospective	162	7	Medial Pivot	Unclassified	preserved
Xiang et al. [36]	2021	Observational Retrospective	1107	5	Medial Pivot	Unclassified	unclassified
Youm et al. [42]	2014	Observational Retrospective	120	5.4	Medial Pivot	Unclassified	sacrificed

Kinematic = true kinematic alignment according to Howell’s surgical technique [51]. Functional = the principle of the technique has been described by the same authors in another study [52]. PCL: posterior cruciate ligament.

**Table 2:** Implants used in the different studies.

Implant	Sample (n)	Percentage (%)	Insert type
Microport Advance	4177	68%	Medial Pivot
Zimmer Persona MC	521	8.5%	Medial Congruent
Bioimpianti K-MOD	417	6.8%	Medial Congruent
Medacta GMK Sphere	291	4.7%	Medial Pivot
Microport Evolution	199	3.2%	Medial Pivot
Finsbury Medial Rotation	172	2.8%	Medial Pivot
Kyocera Phisio Knee	162	2.6%	Medial Congruent
MatOrtho Saiph	126	2%	Medial Pivot
Fine Knee	45	0.7%	Medial Congruent
Kyocera Bi-Surface	33	0.5%	Medial Congruent

1. Advance Medial Pivot, MicroPort Orthopaedics, Arlington, TN, USA; 2. Persona MC (Medial Congruent), Zimmer-Biomet, Warsaw, IN, USA; 3. K-MOD (Knee-Modular Solution), Gruppo Bioimpianti, Peschiera Borromeo (Milan), Italy; 4. GMK Sphere, Medacta, Frauenfeld, Switzerland; 5. Evolution MicroPort Orthopaedics, Arlington, TN, USA; 6. Medial Rotation Knee, Finsbury Orthopaedics, Leeds, UK; 7. Physio Knee, Kyocera, Kyoto, Japan; 8. SAIPH knee system, MatOrtho, Leatherhead, UK; 9. FINE knee, Teijin Nakashima Medical, Okayama, Japan; 10. Bi-Surface TKA with Medial Pivot insert, Kyocera, Kyoto, Japan.

**Table 3:** Summary of alignments.

Alignment	MP Studies	MC Studies	MP Implants (%)	MC Implants (%)
Mechanical	17	7	1653 (55.3%)	916 (30.6%)
Kinematic	3	1	190 (6.4%)	64 (2.1%)
Functional	0	1	0 (0%)	165 (5.2%)

MP = medial pivot, MC = medial congruent. Kinematic = true kinematic alignment according to Howell surgical technique [51]. Functional = the principle of the technique has been described by the same authors in another study [52].

**Table 4:** Clinical outcome average scores. Comparison between medial pivot and medial congruent.

	Sample Total	Score Total	Sample MP	Score MP	Sample MC	Score MC
ROM pre-operative (RANGE, SD)	5447	101.4 (125.9–78.9, SD 9.9)	4434	100.6 (78.9–115.7, SD 9)	1013	105.1 (89.2–125.9, SD 11.7)
ROM post-operative (RANGE, SD)		115.2 (104–132.1, SD 5.8)		114.3 (104–132.1, SD 5.6)		118.9 (108.7–125.8, SD 5.3)
kKSS pre-operative (RANGE, SD)	5202	37.1 (21.3–67.1, SD 8.1)	4496	37.4 (23.5–67.1, SD 7.6)	706	37.4 (21.2–63, SD 10.6)
kKSS post-operative (RANGE, SD)		88 (72.2–96.2, SD 3.7)		87.6 (72.2–96.2, SD 3.9)		88 (72.2–96.2, SD 3.7)
fKKS pre-operative (RANGE, SD)	4305	39.4 (29.7–53.6, SD 3.8)	3764	38.8 (29.7–53.6, SD 4)	541	41.4 (39.6–47.4, SD 2.2)
fKKS post-operative (RANGE, SD)		80.5 (72.4–93.1, SD 4.7)		80.2 (72.4–93.1, SD 4.8)		82.5 (74.3–91.4, SD 5.4)
OKS pre-operative (RANGE, SD)	1601	17 (9.2–23.6, SD 1.8)	1091	16.7 (9.2–23.2, SD 2)	510	17.3 (13.7–23.6, SD 1.6)
OKS post-operative (RANGE, SD)		38.3 (34.9–44.3, SD 1.9)		38.2 (34.9–44.3, SD 2.2)		38.3 (35.8–41.5, SD 1.5)
WOMAC pre-operative (RANGE, SD)	3102	63.2 (34–74.7, SD 6.4)	2589	67 (34–74.7, SD 6.7)	513	46.4 (40.9–48.9, SD 1.84)
WOMAC post-operative (RANGE, SD)		17.4 (5.3–26.8, SD 1.6)		18.4 (6.5–26.8, SD 1.5)		10 (5.32–12.2, SD 0.7)
FJS (RANGE, SD)	3322	69.8 (56.9–96.9, SD 7.8)	2599	69 (56.9–96.9, SD 8.2)	723	72 (60.3–85.7, SD 6.6)

kKSS: Knee Society Score, fKKS: function Knee Society Score, OKS: Oxford Knee Score, FJS: Forgotten Joint Score, WOMAC: Western Ontario and McMaster University score, SD: standard deviation, MP: medial pivot, MC: medial congruent.

**Table 5:** Clinical outcome average scores. Comparison between alignment options.

	Sample F	Outcomes F	Sample M	Outcomes M	Sample K	Outcomes K
ROM pre-operative (RANGE, SD)	/	/	2351	102.7 (78.2–125.9, SD 12.5)	152	115.2 (114.5–115.7, SD 0.3)
ROM post-operative (RANGE, SD)		/		116.5 (104–125.83, SD 6.4)		126.5 (118.7–132.4, SD 8.7)
kKSS pre-operative (RANGE, SD)	/	/	1855	37.5 (21.3–64, SD 9.9)	254	49.4 (42–57.1, SD 4.8)
kKSS post-operative (RANGE, SD)		/		89.3 (82.9–92.4, SD 2.9)		93.8 (88.2–96.2, SD 3.6)
fKKS pre-operative (RANGE, SD)	/	/	1690	42.2 (31.4–53.6, SD 4.1)	166	44.8 (44.1–45.2, SD 0.4)
fKKS post-operative (RANGE, SD)		/		80.9 (72.4–89.5, SD 5.1)		89.3 (84.3–91.4, SD 4.1)
OKS pre-operative (RANGE, SD)	165	21 (21–21, SD 0)	738	17.4 (13.6–23.6, SD 2.1)	47	23.2 (23.2–23.2, SD 0)
OKS post-operative (RANGE, SD)		41.5 (41.5–41.5, SD 0)		38.4 (35.8–43, SD 2.1)		37.9 (37.9–37.9, SD 0)
WOMAC pre-operative (RANGE, SD)	/	/	1230	55.1 (34–74.7, SD 7.4)	47	49.1 (49.1–49.1, SD 0)
WOMAC post-operative (RANGE, SD)		/		13.3 (1.6–25, SD 2.2)		12.5 (12.5–12.5, SD 0)
FJS (RANGE, SD)	165	73 (73–73, SD 0)	1539	72.9 (66–96.9, SD 8.8)	254	70.3 (60.3–89.6, SD 12.8)

kKSS: Knee Society Score, fKKS: function Knee Society Score, OKS: Oxford Knee Score, FJS: Forgotten Joint Score, WOMAC: Western Ontario and McMaster University score, SD: standard deviation, F: functional alignment, K: kinematic alignment, M: mechanical alignment.

**Table 6:** Summary of medial pivot studies.

Author	Year	Sample (n)	Mean Follow Up (y)	Insert Type	Alignment	Outcomes: Pre-Operative/Post-Operative	PCL
Anderson et al. [29]	2010	238	5.4	Medial Pivot	Unclassified	kKSS: 33/90	unclassified
Bae et al. [19]	2011	137	3.9	Medial Pivot	Mechanical	kKSS: 59.6/91.5 fKSS: 53.6/85.4	preserved: 67 sacrificed: 70
Batra et al. [15]	2021	53	4	Medial Pivot	Mechanical	OKS: 9.2/44.3	sacrificed
Brinkman et al. [37]	2014	50	10	Medial Pivot	Mechanical	kKSS: 33.5/84 fKSS: 50/80 WOMAC: 34/22	preserved: 27 sacrificed: 23
Chang et al. [17]	2021	45	2	Medial Pivot	Mechanical	kKSS: 48.2/82.9 fKSS: 48.9/76.2 OKS: 21.9/42.7	sacrificed
Chinzei et al. [44]	2014	85	7.8	Medial Pivot	Mechanical	kKSS:36.2/92.1 fKSS: 31.4/73.4	sacrificed
Choi et al. [32]	2017	49	5.3	Medial Pivot vs. Rotating Platform Mobile Bearing	Mechanical	kKSS: 40.6/89.4 fKSS: 51.9/88.8 WOMAC: 59.1/14.8	sacrificed
Dehl et al. [33]	2018	50	9.5	Medial Pivot	Unclassified	kKSS: 60.7/90.3 fKSS: 48.5/104.4	unclassified
Ettinger et al. [47]	2024	98	2	Medial Pivot	Mechanical vs. Kinematic	kKSS: 54.9/90.3 fKSS: 43.85/81.1 WOMAC: 50.8/12.6 OKS: 22.8/38.3 FJS: -/61	unclassified
Fan et al. [34]	2010	58	5.4	Medial Pivot	Mechanical	kKSS: 30.5/91 fKSS: 36.7/82.3	sacrificed
Hu et al. [21]	2023	252	8.7	Medial Pivot	Mechanical	kKSS: 23.5/89.7 fKSS: 34.3/72.4 WOMAC: 74.4/12.2 FJS: -/77.7	preserved: 84 sacrificed: 168
Indelli et al. [6]	2020	50 vs. 50	2	Medial Congruent vs. Medial Pivot	Mechanical	kKSS: 64/84 fKSS: 45/75 OKS: 20/38	sacrificed
Karachalios et al. [38]	2016	251	13.4	Medial Pivot	Unclassified	kKSS: 31.6/89.2 fKSS: 42.9/78.4 WOMAC: 65.2/26.8 OKS: 15.6/34.9	preserved: 183 sacrificed: 68
Katchky et al. [31]	2019	81	5.3	Medial Pivot	Mechanical	WOMAC: 44.6/6.5	unclassified
Kim et al. [43]	2017	182	12.1	Medial Pivot	Mechanical	kKSS: 29.1/90 fKSS: 44.8/80 WOMAC: 61/25	sacrificed
Kulshrestha et al. [16]	2020	36	2	Medial Pivot	Mechanical	FJS: -/77.9	unclassified
Macheras et al. [45]	2017	347	15.2	Medial Pivot	Unclassified	kKSS: 32.5/92.2 fKSS: 42.7/82 WOMAC: 65.2/16.7 OKS: 15.5/38	preserved: 184 sacrificed: 163
Malhotra et al. [50]	2021	36	3.7	Medial Pivot	Mechanical	kKSS: 46.8/86 fKSS: 42.8/86.5 OKS: 13.6/42.1	sacrificed
Mannan et al. [30]	2009	172	6	Medial Pivot	Unclassified	kKSS: 47.6/72.2 fKSS: 45.1/93.1	unclassified
Schmidt et al. [40]	2014	365	5.3	Medial Pivot	Unclassified	kKSS: 67.1/95.5	sacrificed
Scott et al. [14]	2022	88	2	Medial Pivot vs. Posterior Stabilized	Kinematic	kKSS: 50.6/96.1 FJS: -/68.3	sacrificed
Shakya et al. [48]	2022	52	7.3	Medial Pivot	Mechanical	kKSS: 34/91.1 fKSS: 43.9/89.5 WOMAC: 59.7/11.5 FJS: -/85.6	sacrificed
Shi et al. [39]	2020	290	6.7	Medial Pivot	Mechanical	FJS: -/68.9	unclassified
Sosio et al. [49]	2023	55	2	Medial Pivot	Kinematic	kKSS: 42/94 fKKS: 45/91 WOMAC: 89.6	sacrificed
Ueyama et al. [46]	2020	257	10.1	Medial Pivot	Unclassified	kKSS: 39.7/87.8 fKKS: 41.5/90.3 FJS: -/59.7	sacrificed
Ueyama et al. [41]	2022	153	5	Medial Pivot	Mechanical	kKSS: 39/87 fKSS: 42/90 FJS: -/66	sacrificed
Vecchini et al. [7]	2023	43 vs. 46	2.1	Medial Congruent vs. Medial Pivot	Mechanical	kKSS: 46.4/86.6 fKKS: 49.1/84 FJS: -/96.9	sacrificed
Vecchini et al. [35]	2012	162	7	Medial Pivot	Unclassified	kKSS: 28.3/73.2 fKSS: 49.1/78.9	preserved
Xiang et al. [36]	2021	1107	5	Medial Pivot	Unclassified	kKSS: 24.5/84.7 fKSS: 29.7/74.6 WOMAC: 72.8/20.1 FJS: -/67.3	unclassified
Youm et al. [42]	2014	120	5.4	Medial Pivot	Unclassified	kKSS: 47.6/87.4 fKSS: 38.6/82 WOMAC: 54.8/18.3	sacrificed

PCL: posterior cruciate ligament, kKSS: Knee Society Knee Score, fKKS: function Knee Society Score, OKS: Oxford Knee Score, FJS: Forgotten Joint Score, WOMAC: Western Ontario and McMaster University score. Kinematic = true kinematic alignment according to Howell surgical technique [51]. Functional = the principle of the technique has been described by the same authors in another study [52].

**Table 7:** Summary of medial congruent studies.

Author	Year	Sample (n)	Mean Follow Up (y)	Insert Type	Alignment	Outcomes: Pre-Operative/Post-Operative	PCL
Budhiparama et al. [20]	2023	66	2.7	Medial Congruent	Mechanical	OKS: 23.6/39.8 FJS: -/76.5	preserved: 33 sacrificed: 33
Cacciola et al. [24]	2020	351	5.5	Medial Congruent	Mechanical	kKSS: 33.4/90.6 fKSS: 39.6/81.7 WOMAC: 48.9/12.2 OKS: 13.7/35.8 FJS: -/67.3	unclassified
Giustra et al. [23]	2022	64	2.4	Medial Congruent	Kinematic	kKSS: 48.4/94.5 fKSS: 45.2/91.4 FJS: -/60.3	preserved: 35 sacrificed: 29
Indelli et al. [27]	2023	79	3.6	Medial Congruent	Mechanical	kKSS: -/89 fKSS: -/82 FJS: -/72	unclassified
Indelli et al. [6]	2020	50 vs. 50	2	Medial Congruent vs. Medial Pivot	Mechanical	kKSS: 63/87 fKSS: 43/78 OKS: 19/41	sacrificed
Iwakiri et al. [28]	2022	162	3.8	Medial Congruent	Mechanical	WOMAC: 40.9/5.32	unclassified
Nakamura et al. [25]	2018	45	2	Medial Congruent vs. Cruciate Retaining	Mechanical	kKSS: 55.1/92.2	preserved
Nishitani et al. [26]	2018	33	2	Medial Congruent vs. Cruciate Retaining	Unclassified	kKSS: 37.4/85.1 fKSS: 42.5/74.3	unclassified
Rajgopal et al. [22]	2023	120	1.5	Medial Congruent	Mechanical	kKSS: 21.3/89 FJS: -/84.5	preserved: 60 sacrificed: 60
Rossi et al. [18]	2024	165	6	Medial Congruent	Functional	kKSS + fKSS: 101/164 OKS: 21/41.5 FJS: 73.8	preserved: 80 sacrificed: 85
Vecchini et al. [7]	2023	43 vs. 46	2.1	Medial Congruent vs. Medial Pivot	Mechanical	kKSS: 50.9/85.9 fKSS: 47.4/87 OKS: 21.4/41.5 FJS: 85.7	sacrificed

kKSS: Knee Society Knee Score, fKKS: function Knee Society Score, OKS: Oxford Knee Score, FJS: Forgotten Joint Score, WOMAC: Western Ontario and McMaster University score. Kinematic = true kinematic alignment according to Howell surgical technique [51]. Functional = the principle of the technique has been described by the same authors in another study [52].

## Abbreviations

The following abbreviations are used in this manuscript:

TKA	total knee arthroplasty
MP	medial pivot
MC	medial congruent
MS	medial stabilized
ROM	range of motion
SR	single radius
MR	multi-radius
PCL	posterior cruciate ligament
kKSS	Knee Society Score
fKKS	Function Knee Society Score
OKS	Oxford Knee Score
WOMAC	Western Ontario and McMaster University score
FJS	Forgotten Joint Score
SD	standard deviation
